# ELISPOT Assays in 384-Well Format: Up to 30 Data Points with One Million Cells

**DOI:** 10.3390/cells4010071

**Published:** 2015-01-29

**Authors:** Jodi Hanson, Srividya Sundararaman, Richard Caspell, Edith Karacsony, Alexey Y. Karulin, Paul V. Lehmann

**Affiliations:** Cellular Technology Ltd, 20521 Chagrin Blvd., Shaker Heights, OH, 44122 USA; E-Mails: jodi.hanson@immunospot.com (J.H.); richard.caspell@immunospot.com (R.C.); edith.karacsony@immunospot.com (E.K.); ayk@immunospot.com (A.Y.K.); pvl@immunospot.com (P.V.L.)

**Keywords:** 384-well, ELISPOT, T cell response, assay miniaturization

## Abstract

Comprehensive immune monitoring requires that frequencies of T cells, producing different cytokines, are measured to establish the magnitude of Th1, Th2, and Th17 components of cell-mediated immunity. Antigen titration provides additional information about the affinity of T cell response. In tumor immunity, it is also advisable to account for determinant spreading by testing multiple epitopes. Efforts for comprehensive immune monitoring would require substantial numbers of PBMC to run the above tests systematically, which in most test cases is limiting. Immune monitoring with ELISPOT assays have been performed, thus far, in a 96-well format. In this study we show that one can increase cell utilization by performing the assay in 384-well plates whose membrane surface area is one third that of 96-well plates. Systematic testing of PBMC for antigen-specific T cell response in the two formats demonstrated that the 384-well assay corresponds to a one-in-three miniaturization of the 96-well assay. The lowest number of cells that can be used in the 384-well format, while allowing for sufficient contact with APC, is 33,000 PBMC/well. Therefore, with one million PBMC typically obtained from 1 mL of blood, a 30 well T cell ELISPOT assay can be performed in a 384-well format.

## 1. Introduction

T-lymphocytes are major players in the body’s defense against cancer and various infections. One of the biggest challenges in monitoring T cell-mediated immunity is that these cells occur in various effector classes, secreting different, and in most cases mutually-exclusive, effector molecules. It has become clear that only if all these classes are assessed, will the true nature of T cell immunity be revealed [1]. Another challenge is that many times a multitude of antigens/peptides are targeted by T cells, and only if these are all tested will one obtain reliable information on the magnitude of T cell immunity. Titrating antigen(s) will reveal the affinity of the T cell response. Comprehensive immune monitoring by ELISPOT, therefore, will include testing a multitude of analytes and test conditions. Frequently, trial protocols require several other cellular assays to be performed in parallel, requiring additional PBMC. Ideally, PBMC are cryopreserved for batch testing, but this involves additional cell loss. For all of the above reasons, the number of PBMC that can be obtained from test subjects is often the limiting factor for cellular immune monitoring in general, and in particular for trials that involve pediatric, geriatric, immunosuppressed or oncologic test subjects.

Presently there is no single assay that can measure all the relevant parameters of T cell immunity. ELISPOT assays reveal frequencies and cytokine signatures of antigen-reactive Th1, Th2, and Th17 cells. Measuring the co-expression of IL-2 and IFN-γ in dual color ELISPOT assays (enzymatic or fluorescent) provides information on the numbers of polyfunctional T cells with the same sensitivity as flow cytometry, but requires fewer cells [2]. Detecting T cells that produce Granzyme B and Perforin within 24 h of antigen exposure *ex vivo* signifies that the CD8+ effector cells were recently activated *in vivo* [3]. In contrast, an absence of Granzyme B and Perforin producing T cells within 24 h *ex vivo*, but with detection of these cytokines 3–4 days after subsequent *ex vivo* antigen stimulation, signifies CD8+ memory cells that were resting *in vivo*, but have cytolytic potential upon reactivation [4]. The concentration of the test antigen can be titrated to reveal the affinity of the T cells for said antigen [5], and measurements of such are particularly important for oncologic and autoimmune trials.

In ELISPOT assays every cell seeded in an assay is tested. Increasing the economy of cell utilization even further, the cells survive ELISPOT testing unaffected, and can be reused in subsequent assays [6,7]. Yet, PBMC numbers are frequently limiting for immune monitoring. In an effort to reduce the number of PBMC used, recent studies have introduced ELISPOT assays in which the cells are first pre-activated with antigen in 384-well and 1536-well plates, and then transferred into regular 96-well ELISPOT plates for establishing the numbers of cytokine producing cells [8]. Since such cell transfers are error prone and labor intensive, we verified whether ELISPOT assays could be performed by plating the PBMC directly in dedicated 384-well ELISPOT plates that have a PVDF membrane surface, similar to 96-well ELISPOT plates. It should be noted that these 384-well plates have a membrane surface area that is one-third that of a regular 96-well plate (as opposed to one-fourth, as one might expect based on the number of wells). Since ELISPOT assays are predicated on the detection of T cells in a monolayer, whereby each T cell needs to make contact with an antigen presenting cell (APC), we hypothesized that 384-well assays could be performed with exactly one-third the amount of reagents, including cell material, as would be required for 96-well assays.

## 2. Experimental Section

### 2.1. Peripheral Blood Mononuclear Cells

The human PBMC used for this study were from healthy donors, selected from the reference database at Cellular Technology Ltd [9]. All donors are HLA typed and pre-characterized for T cell activity. The cells were thawed according to an optimized thawing protocol [10]. Briefly, cryovials were stored in nitrogen vapor until being placed in a 37 °C glass bead bath for 10 min (CTL-BB-001, CTL, Cleveland, OH, USA). The cells were diluted slowly with pre-warmed 37 °C Anti-Aggregate medium from CTL (Cat # CTL-AA-005, CTL, Cleveland, OH, USA). PBMC were then centrifuged for 10 min, the supernatant was decanted, the cell pellet was tapped to re-suspend, and then Anti-Aggregate medium was added. A sample of the cells was then counted to determine viability using the Live/Dead/Apoptotic cell counting platform by CTL (Cat# CTL-LDAC-100). Once counted, cells were centrifuged again and then re-suspended in appropriate volumes of CTL-Test Medium (Cat# CTLT-005, CTL, Cleveland, OH, USA) in order to plate the number of human PBMC for each assay as specified.

### 2.2. Human IFN-γ ELISPOT Assays

Both the 384- and the 96-well ELISPOT assays were performed using human IFN-γ kits from Cellular Technology Ltd. (Cat # hIFNG-1M/5 for 96-well kit, and Cat # hIFNG-3M/5 for 384-well kit) Plates and all antibodies, tertiaries, diluents and substrate are contained in the kits. For both plate types, the assay was performed as per manufacturer’s instructions. Briefly, the plates were coated with Human IFN-γ Coating Antibody overnight. The next day, plates were washed once with PBS and then HCMVpp65 antigen (Cat # CEF32-07-005) was plated in CTL-Test Medium. After plating the PBMC in 100 µL for the 96-well plate, or 33 µL for a 384-well plate, the plates were placed in a humidified incubator at 37 °C with 7% CO_2_. After 24 h of incubation, the PBMC were removed, and the detection antibody and development reagents from the kit were added. Following completion of the ELISPOT assay, the plates were air dried in a laminar flow hood prior to analysis.

ELISPOT plates were scanned and analyzed using an ImmunoSpot^®^ S6 Ultimate Reader (S6ULT9000, CTL, Cleveland, OH, USA). Spot Forming Units (SFU) were automatically calculated by the ImmunoSpot^®^ Software for each antigen stimulation condition and the medium (negative) control using the SmartCount™ and Autogate™ functions [11].

### 2.3. Fluorescence Detection of PBMC

PBMC were stained with Calcein AM (C3100MP, Invitrogen, Carlsbad, CA, USA) by incubating 1.0 × 10^6^ PBMC per mL in CTL-Test Media with 1 µL of 1 mM Calcein AM dye in DMSO (Sigma, St. Louis, MO, USA). Following incubation, cells were centrifuged for 10 min and the supernatant was discarded. The cells were washed two times in CTL-Test media and then used in the study.

Fluorescence measurements were performed using the ImmunoSpot^®^ S6 Ultimate Reader (S6ULT9000 CTL, Cleveland, OH, USA) by activating the 480 nm excitation wavelength, and detecting using the 520 ± 20 nm emission wavelength filter.

## 3. Results and Discussion

### 3.1. CD8+ Cell-Derived IFN-γ ELISPOTs Show Identical Size Distributions in 96-Well and 384-Well Assays

**Figure 1:**
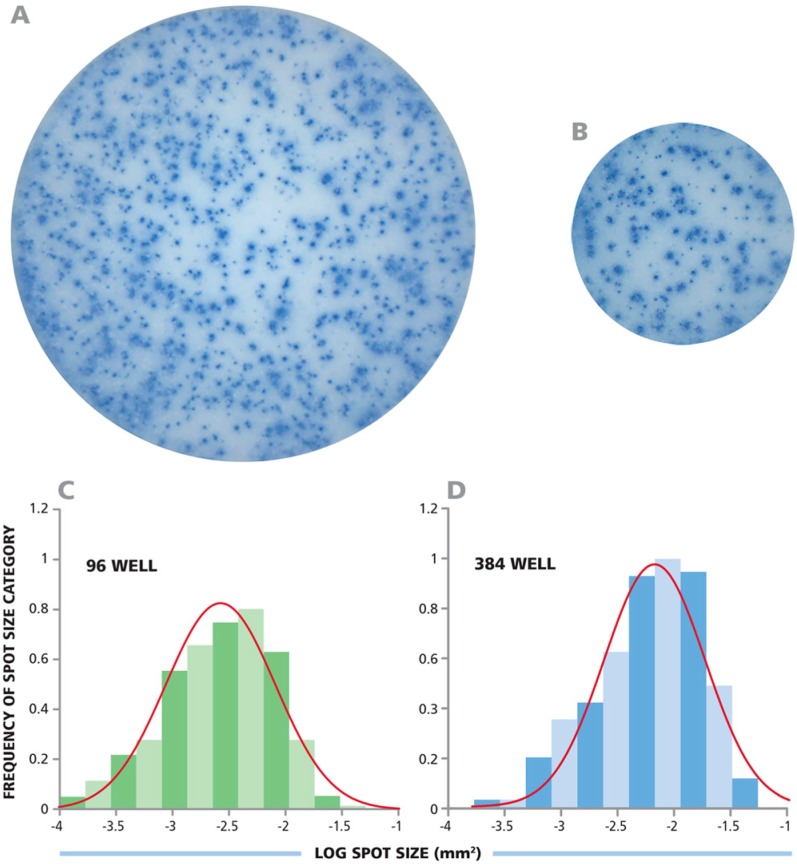
Identical IFN-γ spot sizes and size distributions in the 96- and 384-well format plates. (**A**,**B**) Respective images from a 96-well and a 384-well plate were captured with identical optical and digital resolution; (**C**,**D**) The spot sizes from both wells are plotted as a histogram with a fitted Log Normal curve overlaid in red.

Human IFN-γ ELISPOT assays were performed in parallel in 96- and 384-well plates, using the CEF peptide pool to stimulate CD8+ cells in PBMC. Since the membrane surface in the 384-well format is one-third of the 96-well format, one-third the number of PBMC, as well as all other assay reagents, was used in the 384-well plate. Figure 1A, B show representative well images from both 96- and 384-well plates. To the eye, spot sizes and densities appear similar. However, in order to compare spot sizes in both well formats more stringently, image analysis was performed. The well images of 96- and 384-well plats were captured with identical optical and digital resolution, permitting similar comparisons. The analysis of spot sizes from both well types showed bell shaped curves with identical means and spread (Figure 1C, D). The average spot sizes for 96-well and 384-well plates were −2.575 and −2.514, on a log scale, with a SD of 0.482 and 0.411 respectively. Thus, the sizes of the spots on both plate types were equivalent, demonstrating that the T cell secretory activity per-cell was not affected by the dimensions of the well.

The spot size distributions in the 384-well plates closely approximated the bell shaped curve characteristic of the Normal (Gaussian) function (Figure 1C, D). To statistically determine whether the curves match Normal distribution, we analyzed the curves using the Kolmogorov-Smirnov goodness of fit test. The target significance level was set as α = 0.05. The *p*-value for the spot sizes in the 96-well plate and 384-well plate were 0.419 and 0.220, respectively. Since both p-values were greater than the target significance value of 0.05, the spot size distribution is qualified as being normally distributed.

Establishing Normal spot size distribution is critical, as this permits objective counting. For 96-well plate format [12], and also for 384-well plates, statistics-based automated size gating can be utilized by setting minimal and maximal spot size gates at 3 Standard Deviations (SD) from the mean spot size. When spot sizes are Normally distributed, objects larger than 3 SD of the mean result (with greater than 99.7% likelihood) from cell clusters, and need to be counted as such by estimating the number of spots (*i.e.*, analyte secreting cells) that make up such a cluster. Spots smaller than 3 SD of the mean represent (with greater than 99.7% certainty) assay artifacts that must be gated out. Using these rules for gating, we compared spot counts obtained from the two plate formats.

### 3.2. In Both the 384-and 96-Well Plate, a Linear Relationship between the Number of PBMC Plated and Spot Forming Units (SFU) Is Seen

**Figure 2:**
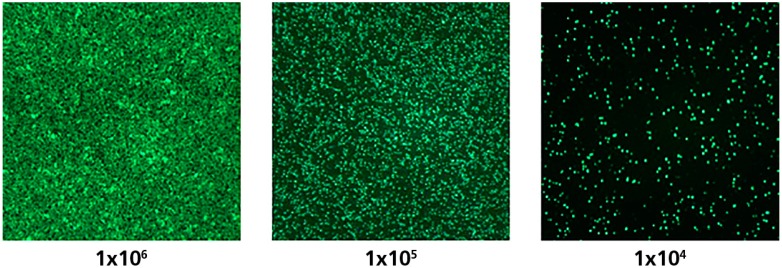
PBMC were stained with fluorescent Calcein AM dye and the specified numbers of cells were plated into respective wells of a 96-well plate. The cells were visualized on an ImmunoSpot^®^ S6 Ultimate Reader by using 480 nm as the excitation wavelength, and detecting fluorescence with a 520 ± 20 nm emission wavelength filter.

For 96-well plates, the relationship between spot numbers and cell numbers was found to be linear between 1.0 × 10^5^ and 1.0 × 10^6^ million PBMC per well—Linearity was not retained above or below these cell numbers [13]. To ensure T cell activation and the resulting cytokine production, T cells and APC must make contact on the membrane. The linear range for cell numbers and spot counts indicates that cell contacts can no longer be made when fewer than 1.0 × 10^5^ PBMC are plated into a well of a 96-well plate, and that cell crowing occurs when more than 1.0 × 10^6^ cells are plated. Between these two extremes, one would expect PBMC to form a monolayer on the membrane, enabling the activation and detection of each antigen-specific T cell. To test this hypothesis, we stained PBMC with a fluorescent dye and plated them into a 96-well plate in a ten-fold serial dilution. As shown in Figure 2, the PBMC are too widely dispersed to enable cell-to-cell contact at 1.0 × 10^4^ cells per well. At 1.0 × 10^5^ cells per well, the PBMC were on the verge of critical density for exhibiting cell-to-cell contacts. At 1.0 × 10^6^ PBMC per well, the cells started to exhibit overcrowding, no longer occurring as a monolayer. Therefore, the linear cell number/spot count data for the 96-well format are consistent with the monolayer hypothesis.

If the monolayer hypothesis is correct, one would expect that plating PBMC at the same cell densities into 384-well plates (*i.e.*, one-third the number of cells in the 3-fold smaller membrane) would also result in a linear spot count to cell number relationship, albeit with one-third the number of spots per well.

**Figure 3:**
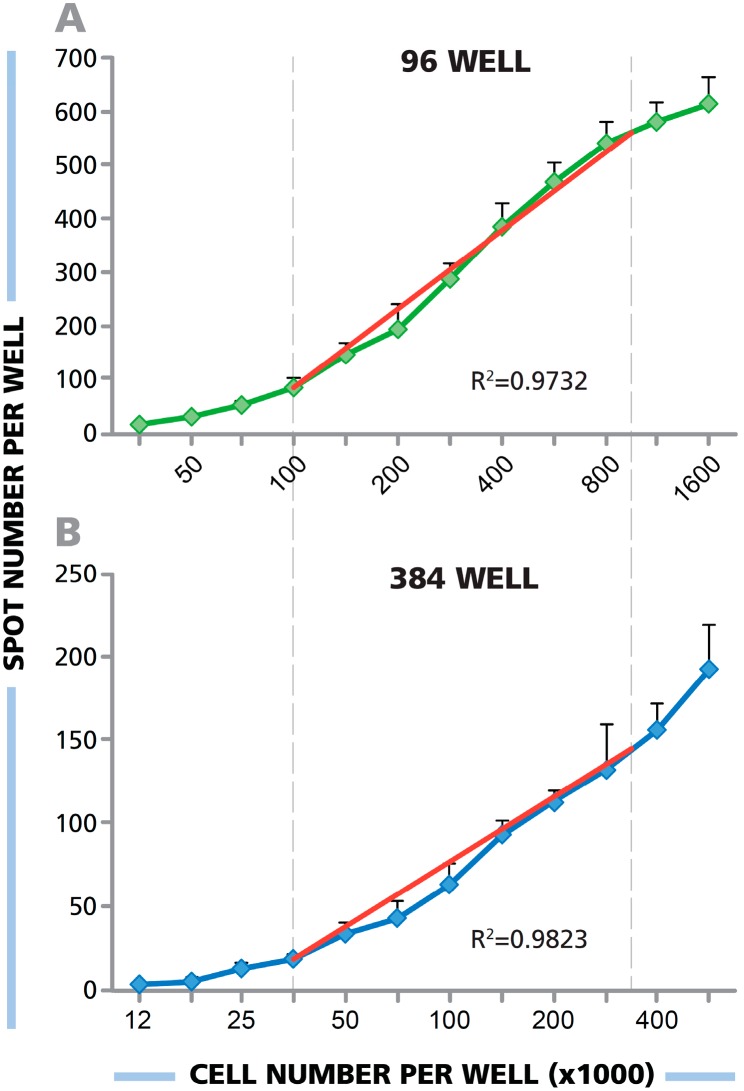
(**A**,**B**) PBMC were plated in an ELISPOT assay in serial dilution at the numbers specified, and HCMV pp65 was added to elicit IFN-γ production by the specific CD8+ cells. The number of PBMC added to a (**B**) 384-well plate was one-third the number in the (**A**) 96-well plate. The ideal linear function is shown by the superimposed red line. For the 96-well plate, the experimental data approximated linearity (R^2^ = 0.9782) between 1.0 × 10^5^ cells per well and 1 × 10^6^ cells per well. For the 384-well plate, linearity was approximated (R^2^ = 0.9823) between 3.3 × 10^4^ PBMC per well and 3.5 × 10^5^ cells per well.

To address the question of cell number/spot count linearity in 384-well format, PBMC were adjusted to 1.5 × 10^7^ cells/mL, and the cells were serially diluted over 12 steps. The cells were plated into 96- and 384-well plates in parallel, pipetting 100μL of the cell suspensions into 96-well plates and 33 μL into the 384-well plate. Subsequently, a human IFN-γ ELISPOT assay was performed using HCMV pp65 antigen to stimulate CD8+ cells. The results are shown in Figure 3. For the 96-well plate, a close to perfect linear relationship was seen between 1.0 × 10^5^ cells per well and 1 × 10^6^ cells per well (R^2^ = 0.9782). For the 384-well plate, there was close to perfect linearity when one-third of these cell numbers were plated, between 3.3 × 10^4^ PBMC per well and 3.5 × 10^5^ cells per well (R^2^ = 0.9823). In both well formats, at higher or lower cell densities than those stated above, spot counts *vs.* cell numbers started to deviate from linearity.

### 3.3. Spot Counts in Replicate Wells of a 384-Well Plate Follow Normal Distribution

Based on the information obtained above, one would expect the spot counts in the 384-well plate to be one-third of the spot counts in the 96-well plate when PBMC are seeded at the same density, *i.e.*, one-third of the absolute numbers per well. Because of the inherent variability between replicate wells, which for 96-well plates was shown to follow a Normal distribution [14], a direct well-to-well comparison between 96-well and 384-well format is best done by establishing whether variability among replicates follows the same rule in 384-well format. Furthermore, the Normal distribution of spot counts in replicate wells allows one to determine whether parametric statistics can be used to compare antigen-induced spot counts with spot counts in the medium control wells, identifying cut-offs for positive responses.

**Figure 4:**
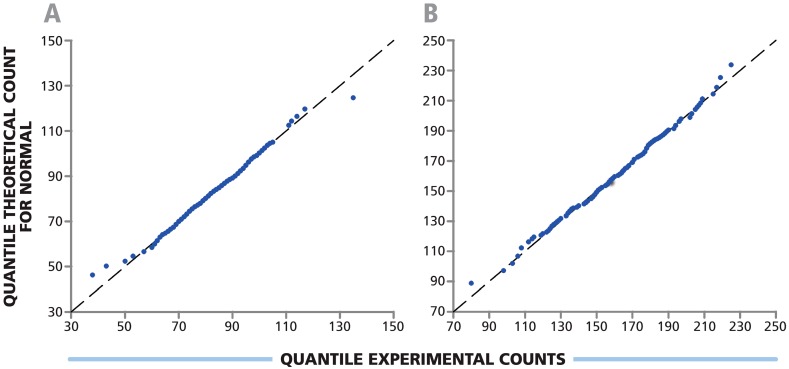
Spot count variation among replicate wells of 384-well plate follows Normal distribution. PBMC were plated at (**A**) 0.5 × 10^5^ cells per well in 384 replicate wells; and (**B**) 1.0 × 10^5^ cells per well in 192 replicate wells. The response to HCMV pp65 antigen in an IFN-γ ELISPOT assay was measured. Normality was determined using Shapiro Wilk test with α value of 0.05 and *p*-values of 0.708 and 0.388 respectively. Q-Q plots for experimental *vs.* theoretical counts are shown.

PBMC were tested in 384-well plates with 384 replicate wells at 5 × 10^4^ cells per well, and with 192 replicates at 1.0 × 10^5^ cells per well. In both instances HCMV pp65 was used to stimulate CD8+ cells. Shapiro Wilk test was done to assess whether the spot counts among the replicate wells follow Normal distribution. According to this test, the *p*-values for both cell dilutions were 0.708 and 0.388, with α = 0.05. Since *p*-values were larger than the significance level (α), spot numbers in replicate wells were considered Normally distributed. Q-Q plots (Figure 4) for experimental spot counts in replicate wells were linear confirming the results of Shapiro Wilk test. This proves that the spot counts in replicate wells of 384-well plates, as previously reported for 96-well plates [14], follow Normal distribution function. Therefore, in 384-well plate as well as 96-well plate assays, parametric statistics (including the Students’t-test or ANOVA) can be used to compare test results. This also means that there is no need for empirical cutoff values (for instance >10 spots for 96-well plates) to determine positive from negative response wells. In past studies, the “minimum number of spots” was defined in order to distinguish positive from negative responses because they could not be subject to parametric statistical testing, which can make these distinctions unambiguously.

### 3.4. SFU in 384-Well Plates Are One-Third Those in 96-Well Plates

We tested 12 different cryopreserved PBMC samples in both plate formats, in parallel, of which 11 samples exhibited a positive response to HCMV pp65. The cells were plated at 3 × 10^5^ PBMC per well in 96-well plates in triplicate, and one-third of that cell number (1.0 × 10^5^ per well) in the 384-well plate in triplicate. For the 384-well plate, all other reagents used were also used at one-third the volumes used in the 96-well plate. The results of both the 96- and 384-well plate assays were counted using the ImmunoSpot^®^ Software (version 5.3.6) which includes a 384-well plate counting suite. As can be seen in Table 1, for all eleven positive donors, the mean number of SFU (of the three replicates per well) in the 96-well plates was approximately three times the number of the mean SFUs in the 384-well plate. When assessing the mean (of triplicate values) of means (for the 11 donors) an almost perfect one-in-three relationship was obtained.

**Table 1:** Spot counts in 96-well format were approximately three times the spot count in 384-well plate format. For 11 test subjects, the HCMVpp65-induced response was measured in triplicate wells for each plate format with the mean of the triplicate spot counts shown. The ratio between these mean spot counts in 96-well format and 384-well format is shown in the right column.

TEST	96-WELL	384-WELL	96W/384W
1	670.0	177.5	**3.8**
2	577.5	177.3	**3.3**
3	547.3	173.8	**3.1**
4	499.3	164.0	**3.0**
5	423.0	118.5	**3.6**
6	344.5	102.8	**3.4**
7	260.0	77.8	**3.3**
8	140.3	52.3	**2.7**
9	126.0	35.5	**3.5**
10	66.8	21.8	**3.1**
11	26.0	7.5	**3.5**
			**MEAN 3.3 ± 0.3**

### 3.5. Matching Dose Response Curves Are Seen in 96- and 384-Well Format

In order to cope with the limited numbers of test subject PBMC available, immune monitoring efforts have primarily relied on measuring T-cell responses at a single dose of antigen. Such measurements, however, miss out on critical information about the T cells’ functional affinity for the antigen, which can be determined by testing serial dilutions of the antigen [5]. Given the three-fold economization of cell utilization with the 384-well format, it should become feasible to include affinity measurements into T cell monitoring assays.

**Figure 5:**
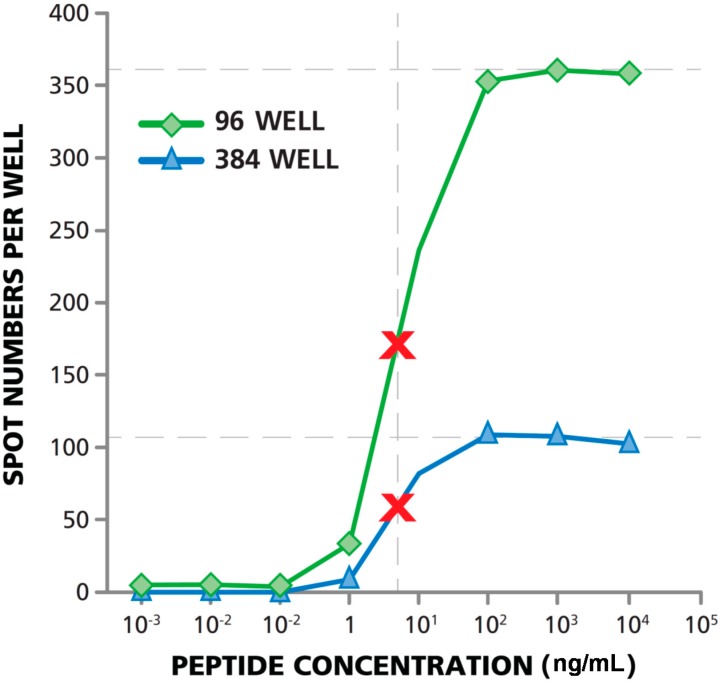
T cell functional affinity measurements in 384- and 96-well formats provide identical results. HCMV peptide pp65-induced IFN-γ production by CD8+ cells was measured in PBMC in both 96-well and 384-well plate types. The highest peptide concentration tested was 10 μg/mL with subsequent 1:10 serial dilutions. The numbers of PBMC plated in the 96-well format was 3 × 10^5^/well (green line), and 1 × 10^5^/well for the 384-well plate (blue line). Fifty percent maximal activation is marked with the red X.

We tested whether T cell functional affinity measurements performed in both 96- and 384-well formats would provide the same results. PBMC were seeded at 3 × 10^5^ cells per well into the 96-well plates, and at 1.0 × 10^5^ cells per well into the 384-well plates. HCMV pp65 antigen was titrated from 10 µg/mL to down to 1.0 × 10^−6^ µg/well. As shown in Figure 5, the dose response curves for both plate types were parallel. The curve reached a plateau at ~360 spots per well in the 96-well plate, and at ~105 spots per well in the 384-well plate. Fifty percent maximal stimulation was therefore 180 spots per well for the 96-well plate, and 53 spots per well for the 384-well plate. The antigen concentration at which 50% maximal stimulation is reached represents the K_eff_ 50 value, defining affinity. The K_eff_ 50 value was close to identical in both well formats, 7.5 ng/mL. The data show that the 384-well assay is just as capable of measuring T cell functional affinity as the 96-well assay, however, it only requires one-third of the number of cells.

Replicate wells are not typically required when antigens are serially diluted, as the serial wells with successive antigen concentrations confirm positive responses. Functional affinity measurements in 384-well format can be done, with the same amount, or fewer, PBMC than a regular 96-well measurement at a single antigen dose. In a regular 96-well assay, testing for antigen-reactivity in triplicate wells at a single antigen dose, with 3 × 10^5^ PBMC per well requires 9 × 10^5^ PBMC plus the same number of PBMC for the medium controls, *i.e.*, 1.8 × 10^6^ PBMC. In corresponding 384-well format, the cells would be seeded at 1.0 × 10^5^ cells per well; with 1.8 × 10^6^ PBMC one could test (assigning three wells for the medium controls) 15 additional wells for testing the dose response curve, obtaining invaluable additional information on the affinity of the T cell response.

### 3.6. Lower Signal-to-Noise Performance of 384-Well Assays

**Figure 6:**
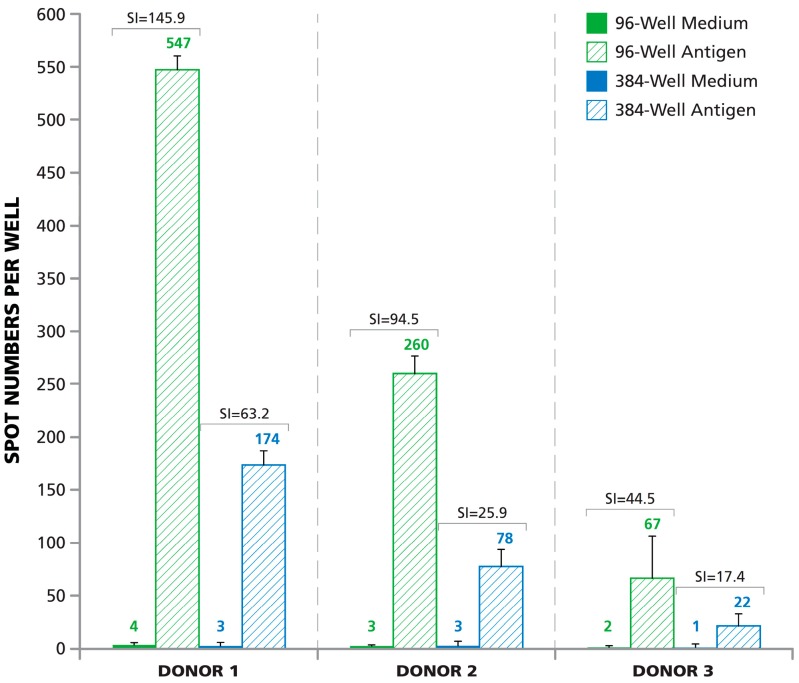
Signal-to-noise performance of 96-well *vs.* 384-well plate. HCMV pp65 antigen-induced responses (hatched bars) and medium controls (solid bars—Mostly too small to be seen) are shown for three donors with high, medium, and low response levels. PBMC numbers were 3 × 10^5^ for the 96-well plate and 1.0 × 10^5^ for the 384-well plate. The mean spot count/well calculated from three replicate wells for each condition is specified above the bars. The stimulation index (SI) is defined as the number of ELISPOTs induced by an antigen divided by the number of spots in the medium background. SI for the corresponding medium control/antigen pairs is specified.

The data thus far showed that a one-in-three down-scaling of PBMC and reagents in an ELISPOT assay in the 384-well format results in a proportional one-in-three reduction of the antigen-induced spot counts, *i.e.*, of the signal. As interpretation of ELISPOT data depends on the signal to noise (media background) ratio, we compared this between the two plate formats. We tested three different cryopreserved PBMC samples that had been previously identified as eliciting a high, medium, or low response respectively to HCMV pp65 antigen in both well formats, in parallel. As expected from the previous results, the number of antigen-induced SFU in the 384-well plate was one-third the number of SFU in the 96-well plate (Figure 6). However, we observed similar numbers of SFU in the medium control wells in both the 96-well and 384-well plates. This number was between 1 and 4 spots for the three donors. The signal to noise ratio for the 96-well plate was on average 2.8-fold (2.3-, 3.6-, and 2.6-fold) higher than for the 384-well plate. The lower signal to noise performance of the 384-well assay must be considered when attempting to identify weak T cell responses. One method to improve signal to noise ratios would be to purify CD8 cells from the PBMC population and use purified CD8 cells as opposed to PBMC, for samples with weak responses. CD8 cells can present antigen to each other and therefore do not require additional non-secreting APCs. Generally, CD8 cells comprise ~25% of PBMC, thereby increasing signal by fourfold, when cell numbers plated are the same. Moreover, since no bystander cells are present in the assay, the noise will also reduce, providing even greater signal to noise ratios.

### 3.7. Low Spot Counts From 384-Well Plate Have a Higher Coefficient of Variation Than Corresponding Wells From 96-Well Plate

In ELISPOT assays rare antigen specific T cells are identified within a vast excess of bystander cells. One would therefore, expect the coefficient of variation (CV: The well-to-well variability among replicates) to increase as the number of specific T cells in the test sample decreases. In other words, the lower the frequency of antigen-specific T cells in a given test sample, the higher the variability of such cells in the defined sample volume pipetted in replicate wells. One would also predict that at the same low concentration of antigen-specific cells, the well-to-well variability will be higher when the sample volume is lower. As 100 μL of PBMC sample is plated in the 96-well assay, and 33 μL of PBMC at the same concentration is plated in the 384-well assay, the CV should be higher for the 384-well plate.

**Figure 7:**
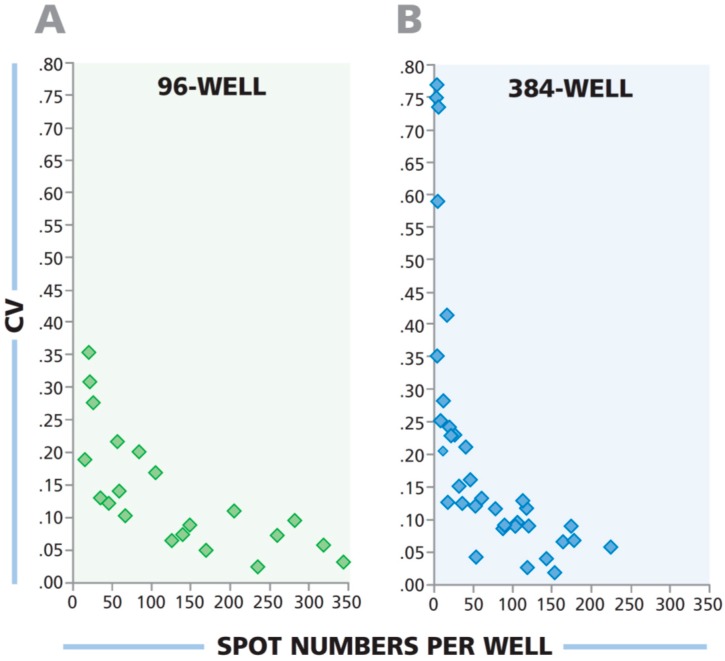
The variability in spot counts among replicate wells is higher in 384-well plates than in 96-well plates when low frequency T cells are being detected. PBMC from three donors were serially diluted and tested, in quadruplicate wells for each donor and cell dilution, for antigen-elicited IFN-γ response. The PBMC were plated in serial dilution between 1.0 × 10^6^ and 1.0 × 10^5^ cells per well in the 96-well plate, and at 3 × 10^5^ and 3 × 10^4^ cells per well in the 384-well plate. The CVs of the quadruplicate wells were plotted against mean spot numbers for the corresponding test results.

To experimentally test these predictions, we performed human IFN-γ ELISPOT assays, stimulating PBMC of three donors with four antigens while serially diluting the PBMC in a linear range to obtain a wide spread of spot counts. Each cell sample and antigen dilution was tested in quadruplicates. In Figure 7, the CV was calculated for each quadruplicate and plotted against the mean spot number for that quadruplicate. For both plate formats, the CV for wells with fewer SFU was found to be larger than for wells with more SFU. The corresponding wells of a 96-well plate exhibited a lower CV compared to the 384-well plate. This notion also needs to be taken into account when testing samples that contain low frequency antigen-specific T cells in the 384-well format.

## 4. Conclusions

ELISPOT assays, when performed in regular 96-well format, already provide far better cell utilization as compared to other T cell monitoring techniques. By introducing 384-well ELISPOT assays, we have been able to reduce the number of PBMC required by three-fold, corresponding to the three-fold reduction in membrane surface area for the 384-well plate. A systematic side by side comparison of the two assay formats showed that antigen-induced spot counts in 384-well format (obtained with one-third of the PBMC numbers per well) will be one-third of those obtained in 96-well format. Thus, high- and moderate-frequency antigen-specific T cells can be readily detected in 384-well format. However, when it comes to low-frequency T cells, decreased stimulation indices and increased CVs among replicates were observed as an inevitable consequence of the fewer PBMC plated. For low-frequency measurements in the 384-well format, increasing the number of replicate wells can compensate for the decreased resolution. As ELISPOT counts among replicate wells follow Normal Distribution in the 384-well format, parametric tests, including Students’ t-test and ANOVA, can be used to identify positive responses. This is especially important for weak responses, as increasing replicate wells can identify weak responses from negative responses with greater statistical significance.
